# Placenta Pathologies in Two Patients With Glycogen Storage Disease Type Ia and Preeclampsia

**DOI:** 10.1002/jmd2.70025

**Published:** 2025-06-12

**Authors:** V. Laufs, A. Hasenburg, M. A. Busch, F. Lang, D. Macchiella, E. Mildenberger, L. Seidmann, J. B. Hennermann

**Affiliations:** ^1^ Clinic for Obstetrics and Women's Health University Medical Center of the Johannes Gutenberg‐University Mainz Mainz Germany; ^2^ Division of Neonatology Center for Pediatric and Adolescent Medicine, University Medical Center of the Johannes Gutenberg‐University Mainz Mainz Germany; ^3^ Villa Metabolica Center for Pediatric and Adolescent Medicine, University Medical Center of the Johannes Gutenberg‐University Mainz Mainz Germany; ^4^ Institute of Pathology University Medical Center of the Johannes Gutenberg‐University Mainz Mainz Germany

**Keywords:** carbohydrate storage disorder, fetal growth restriction, GSD Ia, histologic features, inherited metabolic disease, placenta abnormalities, preeclampsia, small for gestational age

## Abstract

Little is known about pregnancies and placental changes in women with glycogen storage disease type Ia (GSD Ia). We report on two primipara with GSD Ia who both developed preeclampsia and whose newborns were small for gestational age. Both placentas showed sonomorphological and macroscopical abnormalities. Disease‐specific histological features could not be identified, which should prompt further research.

**Summary**

- Two pregnant women with glycogenosis type Ia developed preeclampsia and had children born SGA. Their placentas showed abnormalities but no specific histological features of GSD Ia. Further research is needed to clarify the effects of metabolic disorders on the placenta and fetal growth.

## 1 Introduction

Glycogen storage disease type Ia (GSD Ia) is a rare (overall incidence ~1/100000) [1] autosomal recessive inherited disorder of carbohydrate metabolism, caused by mutations in the *G6PC gene* that result in a deficiency of glucose‐6‐phosphatase. Patients typically present during the first year of life with fasting intolerance, recurrent hypoglycemia, and hepatomegaly. Long‐term complications are growth retardation, hepatic adenoma, risk of hepatocellular carcinoma, renal and ovarian cysts, chronic kidney disease, and osteoporosis. Biochemical derangements include, besides hypoglycemia, elevated transaminases, hyperlactatemia, hyperuricemia, and hyperlipidemia.

Current treatment of GSD Ia consists of a carbohydrate‐based diet with 60%–70% of energy coming from carbohydrates, avoidance of simple sugars, frequent meals including intake of uncooked cornstarch during the day and once to twice during the night. The use of complex carbohydrates allows for stable glucose control and reduces hypoglycemia and hyperglycemia as recommended for GSD Ia [2]. Frequent blood glucose monitoring, for example, glucose monitoring systems (CGM, continuous glucose monitoring), is required to maintain blood glucose within normal concentrations and to prevent metabolic decompensations as well as long‐term complications [3].

As life expectancy in these patients has improved, pregnancy becomes more and more an issue. However, only a few pregnancies in women with GSD Ia have been reported [4, 5, 6, 7, 8, 9, 10, 11]. We here report the course of pregnancy and delivery in two primipara with GSD Ia and discuss whether special pathological changes of the placenta may have an impact on SGA (small for gestational age)/FGR (fetal growth restriction) development in pregnancies of GSD Ia mothers.

## 2 Case Series

### 2.1 Patient 1

The first patient, 26 years of age, had been diagnosed at the age of 9 months with GSD Ia due to hepatomegaly and hyperlactatemia. Dietary treatment was initiated at the age of 9 months, and the patient was provided with a percutaneous gastrostomy for enteral nocturnal feeding from the 1st until the 14th year of life. At the age of 13 years, intake of Glycosade, a hydrothermally processed high amylopectin cornstarch, was implemented in the dietary regimen. Due to hepatocellular carcinoma, focal nodular hyperplasia, and multiple liver adenomas, she underwent two partial liver resections at the age of 17 and 24. Further long‐term complications of GSD Ia were ovarian cysts, renomegaly with hyperfiltration, hyperuricemia, hyperlactatemia, hypertriglyceridemia, and iron deficiency. Furthermore, she suffered from Hashimoto's thyroiditis, cholecystolithiasis, and recurrent gastritis. Her therapy of GSD Ia consisted of frequent meals, intake of uncooked cornstarch, and one dose of Glycosade during the night. Adherence to diet was poor, and the patient refused the use of CGM for metabolic control. The patient was listed for liver transplantation at the age of 25 years, with current status “NT” (not transplantable).

The patient had a history of two early abortions at 9 and 5 weeks of gestation. During the 3rd pregnancy, she first presented in our clinic at 16 + 4 weeks of gestation. Laboratory control at baseline, that is, first measurement during pregnancy, revealed normal serum concentrations of uric acid (5.1 mg/dL) and lactate (1.8 mmol/L) with a slight increase of serum triglycerides (230 mg/dL). As a parameter for hyperfiltration, eGFR was increased up to 147 mL/min/1.73 m^2^. At the 20th week of gestation, CGM was started, with consistently adapting carbohydrate intake to the current blood glucose level. Metabolic control during the 2nd and 3rd trimester of pregnancy revealed hyperlipidemia, hyperfiltration, albuminuria, and proteinuria. Blood glucose and lactate values recorded at outpatient visits during the 2nd and 3rd trimester of pregnancy were within the recommended range between 79 and 89 mg/dL for glucose and between 1.6–1.8 mmol/L for lactate. Data from CGM, performed during the 25th week of pregnancy, revealed 1–3 hypoglycemias, defined as blood glucose levels below 70 mg/dL, per day but also recurrent postprandial glucose concentrations of > 180 mg/dL. Serum triglyceride concentrations ranged between 230 and 349 mg/dL. Hepatic adenomas did not enlarge during pregnancy.

**FIGURE 1:**
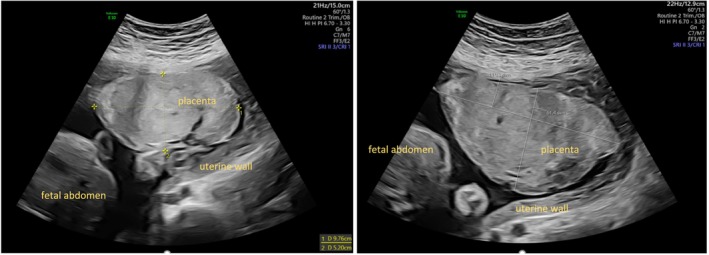
Ultrasound placenta case 1 (29 + 3 and 31 + 3 weeks of gestation). The placenta has a small adhesive surface and is thickened.

At her first visit during pregnancy, there were normal ultrasound findings with an estimated fetal body weight of 181 g (75th percentile). At 20 weeks of gestation, ultrasound revealed a placenta of small size with high thickness (Figure 1). Uterine artery doppler assessment revealed elevated indices. At 29 weeks of gestation, umbilical arterial doppler showed elevated indices as well. In addition, mild proteinuria (500 μg/mg; norm < 200 μg/mg, protein/creatinine ratio) was detected. As fetal growth restriction of the abdominal circumference was identified at 30 weeks of gestation, the patient was admitted to hospital for closer surveillance. The patient reported increasing contractions and fluctuating blood glucose concentrations. Prenatal steroids and tocolysis with TRACTOCILE (atosiban) were administered. During this time, the cardiotocogram repeatedly showed variable decelerations. The patient developed signs of preeclampsia, that is, increasing proteinuria (1304 μg/mg, norm < 200 μg/mg, protein/creatinine ratio), slightly increased blood pressure (max. 145/83 mmHg), a discreet elevation of GOT (glutamate oxaloacetate transaminase) (36 U/L), headache, and severe peripheral oedema. Therefore, cesarean section was performed at 31 + 5 weeks of gestation. A peripartum i.v. treatment was started with an electrolyte‐glucose infusion with 3.46 mg glucose/kg/min on continuous glucose and lactate monitoring.

A male infant was born with a weight of 1290 g (9th percentile), an Apgar score of 5/5/8, and an umbilical artery pH of 7.32. The child was admitted to the neonatal intensive care unit and received routine supportive treatment according to the gestational age. Parenteral carbohydrate intake was 8 g/kg/day at maximum, increasingly replaced by enteral nutrition until reaching full enteral feeding at day 10 of life. No alterations of blood glucose and lactate were recorded. The postnatal course was uneventful.

**FIGURE 2:**
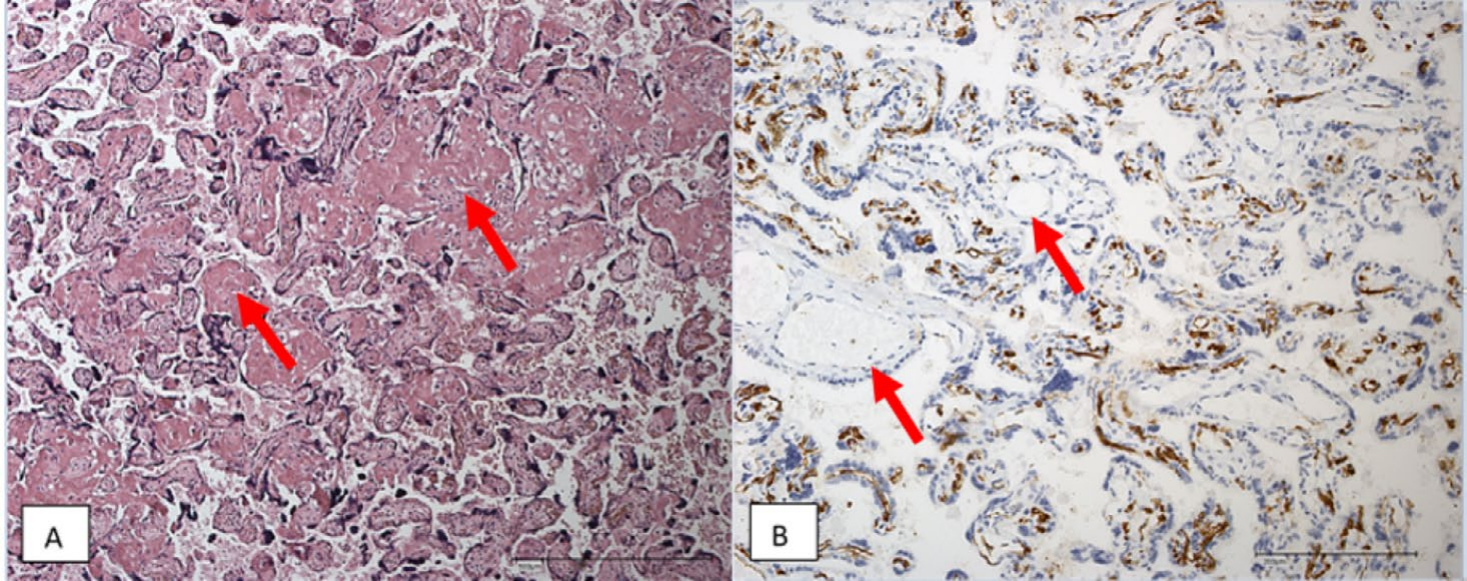
Histological findings case 1. (A) Acceleration of villous maturation (HE, ×100, arrows: Perivillous microfibrin deposits). (B) Appropriate differentiation of the feto‐placental endothelium: Negativity for CD15 in vessels of stem villi (arrows) and positivity in many capillaries of a focus of terminal villi (CD15 immunoreactivity in placental tissue, ×200).

The placenta weighed 275 g (< 10th percentile) and was 12.5 × 10.0 × 2.2 cm in size. Histological examination revealed non‐specific patterns of regional malperfusion of the maternal and fetal vessels (Table 1, Figure 2). Morphologically, there were no signs of severe chronic placental insufficiency or fetal hypoxia.

**TABLE 1:** Pathomorphological placenta changes in pregnancies with GSD 1a, PE, and GDM [7, 12, 13, 14, 15, 16, 17].

Patterns of placental pathology	Stigmata	GSD Ia			GDM	PE
		Case 1	Case 2	Сase report [7]		
Placenta weight [12, 14]	Small placenta	X	—	X		(X)
	Large placenta	—	X	—	(X)	
Maturation defect [12, 13]	Arrest of villous maturation and persisting embryonic stroma	—	X	—	(X)	—
Maternal vascular malperfusion [14]	Accelerated villous maturation	X	—	—	—	(X)
	Infarction	—	—	X	(x)	(X)
	Inter‐ and perivillous fibrin deposition (pseudoinfarction)	X	x	—	(x)	(X)
	Trophoblastic knots	X	x	—	(x)	(X)
	Subchorionic pseudoinfarction with calcification	x	X	x	(x)	(x)
Fetal vascular malperfusion [14]	Fetal vasculopathy associated with calcification	x	x	—	(x)	(x)
Metabolic storage diseases [15]	Trophoblast/macrophage vacuolation	—	—	—	—	—
Fetal hypoxia [16, 17]	Fetal erythroblastosis	—	—	—	(x)	(x)
	Macroendothelial immaturity	—	x	—	(x)	(x)
	Meconium exposure	—	—	—	(x)	(x)

Abbreviations: GDM, gestational diabetes mellitus; GSD Ia, glycogen storage disease type Ia; PE, preeclampsia; x, minor finding (< 5% of placental villi), typical findings in GDM and PE are in brackets; X, major finding (> 5% of placental villi).

### 2.2 Patient 2

The second patient, 33 years of age, was diagnosed postnatally with GSD Ia due to a positive family history. Dietary treatment was initiated immediately after diagnosis and included cornstarch feeding during the night. She never received a percutaneous gastrostomy. She had undergone multiple partial liver resections due to progressive liver adenomatosis at the ages of 25, 28, and 30. Long‐term complications of GSD Ia were liver fibrosis, hyperuricemia, hyperlactatemia, hypertriglyceridemia, hypercholesterolemia, and osteopenia. Furthermore, she suffered from essential arterial hypertension, which was well controlled with antihypertensive agents. She used uncooked cornstarch to control GSD. Adherence to the diet was poor, and the patient refused the use of CGM for metabolic control. The patient was listed for liver transplantation at the age 26 years, with current status “NT”.

The patient had a history of one ectopic pregnancy. During her 2nd pregnancy, she presented in our clinic at 8 + 1 weeks of gestation. Laboratory control at baseline, that is, first measurement during pregnancy, revealed an increase of serum triglycerides (347 mg/dL), uric acid (8.3 mg/dL), and lactate (5.7 mmol/L). CGM with adaptation of dietary treatment was started at the 12th week of gestation. Metabolic control during pregnancy revealed hyperlactatemia, severe hypertriglyceridemia (498–861 mg/dL) and hyperfiltration (eGFR 140 mL/min/1.73m^2^). Glucose and lactate values recorded at outpatient visits during the second and third trimester were fluctuating and partially outside the recommended range, with serum glucose concentrations between 66 and 119 mg/dL and lactate concentrations between 2.7 and 5.7 mmol/L. On CGM, fluctuating blood glucose concentrations were reported. Hepatic adenomas did not enlarge during pregnancy.

**FIGURE 3:**
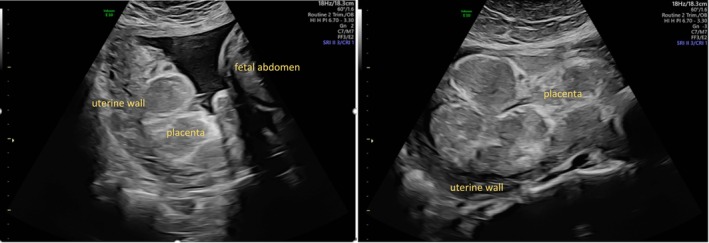
Ultrasound placenta case 2 (31 + 6 weeks of gestation). Significant calcifications appear early on in pregnancy.

At first presentation, ultrasound findings were normal. At 27 + 2 weeks of gestation, estimated fetal weight was below the 3rd percentile and the placenta showed signs of abnormality with a pronounced pattern of calcifications (Figure 3). Uterine artery doppler measurements were normal. In week 33 + 1, the amniotic fluid volume was above average but stabilized on a high‐normal basis. At 35 + 3 weeks of gestation, the patient presented at the hospital due to elevated blood pressure and headache. A cesarean section was performed due to the aggravation of preeclampsia. A peripartum i.v. treatment was started with an electrolyte‐glucose infusion with 3.46 mg glucose/kg/min on continuous glucose and lactate monitoring.

A female infant was born weighing 2015 g (9th percentile), with an Apgar score of 6/8/8 and an umbilical artery pH of 7.28. For blood glucose and lactate monitoring, the child was admitted to the neonatal intensive care unit. Parenteral carbohydrate intake was started with 8 g/kg/day, increasingly replaced by enteral nutrition until reaching full enteral feeding at day 4 of life. No alterations of blood glucose and lactate were recorded, and the postnatal course of the newborn was uneventful.

**FIGURE 4:**
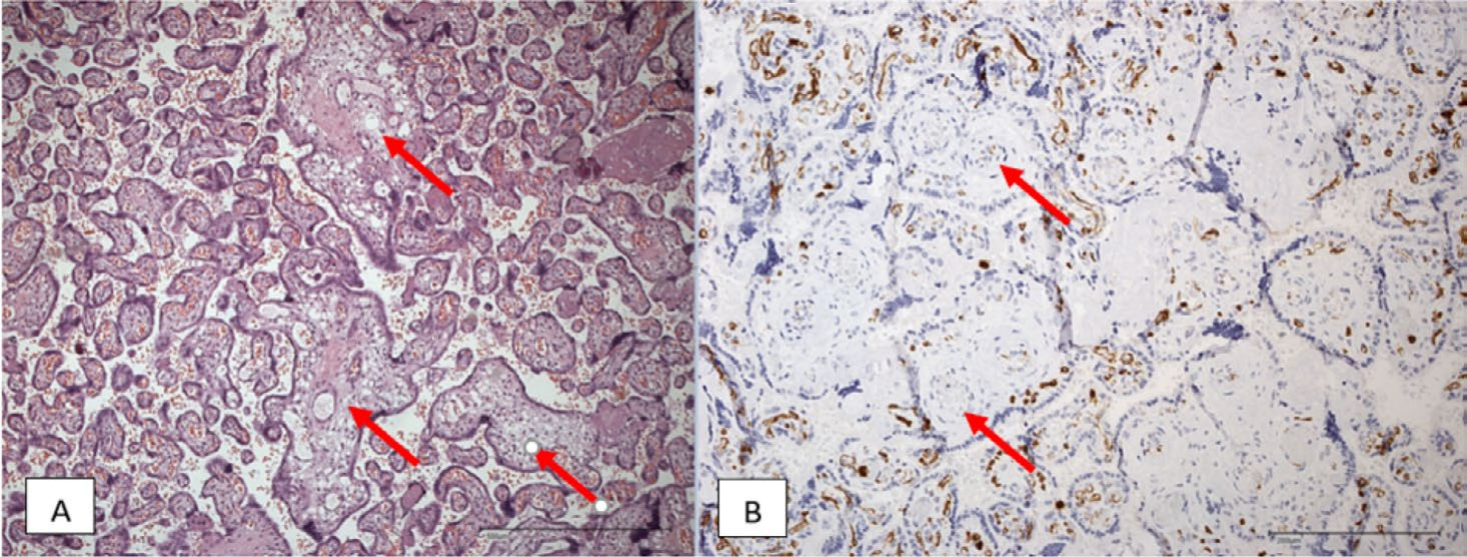
Histological findings case 2: (A) Maturation defect with focal villous arrest (arrows) and perivillous fibrin plaque (pseudo infarctions) as found in gestational diabetes (HE, ×100). (B) Appropriate differentiation of the feto‐placental endothelium, negativity for CD15 in vessels of stem villi (arrows) and positivity in capillaries of terminal villi (CD15 immunoreactivity in placental tissue, ×200).

The placenta weighed 550 g (> 90th percentile) and was 18.0 × 15.0 × 3.5 cm in size. Histology showed dissociated villi maturation disorders as a symptom of chronic insufficiency of the placenta. Histological examination confirmed pseudoinfarctions with calcifications showing a regional utero‐placental malperfusion in the course of clinically suspected preeclampsia (Table 1, Figure 4).

## 3 Discussion

Only a few reports on pregnancies in women with GSD Ia are available, and little has been reported about specific changes of the placenta in GSD Ia pregnancies. There is one case report of a triplet pregnancy. As in our patients, the patient developed preeclampsia at 32 weeks of gestation, and as her condition worsened, a cesarean section was performed in the 35th week of pregnancy. One of the children showed FGR (birth weight 2nd percentile), but in this case, more likely due to fetofetal transfusion syndrome in bichorionic triamniotic gestation [18]. In line with our observation, Lee et al. reported a case of otherwise unexplained growth restriction [9]. Yamamoto et al. described a case of GSD Ia complicated by fetal growth restriction and preeclampsia [7]. Due to hypertension and FGR, a cesarean section was performed at 26 weeks of gestation. Of note, there are several case reports of GSD Ia without fetal growth restriction (Table 2).

**TABLE 2:** Summary of clinical information, maternal and fetal pregnancy outcomes from cases reported in the literature.

References	Clinical information	Maternal pregnancy outcome	Neonatal outcome	FGR yes/no
Martens, Rake et al. 2008 [4]	**15 pregnancies** − In 6/15 increase in frequency and/or severity of hypoglycemia − Carbohydrate requirements varied intra‐ and inter‐individually − 1 patient developed hypertriglyceridemia in second trimester − No evidence for increase in size or number of adenomas	− In 12/15 cesarian section; various reasons not related to GSD − 1 patient developed intermittent hypoglycemia and mild lactate acidosis during delivery − 1 patient severe hyperlactatemia and metabolic acidosis during delivery despite high‐normal glucose levels	− 2 cases of prematurity − 1 case of FGR − 1 case of macrosomia − 4/15 neonates showed hypoglycemia − Normal psychomotor development at age of 1 year in 14/15 children (delayed development in one of the premature children)	Yes (1/15)
Sechi, Deroma et al. 2013 [5]	**5 pregnancies in 4 patients** − Increase in frequency and/or severity of hypoglycemia in 1 woman − Increase in size in one pre‐existing adenoma, in other patient no change of pre‐existing adenomas	− 2 preterm cesarean deliveries (fetal distress due to placenta praevia, maternal distress due to lung infection) − 2 term cesarean deliveries (to prevent hypoglycemia, breech position)	− All children normal psychomotor development	No
Mairovitz, Labrune et al. 2002 [6] Ryan, Havel et al. 1994 [10]	− **Case 1** ^a^: singleton pregnancy, uneventful pregnancy − **Case 2** ^a^: triplet pregnancy; frequent episodes of hypoglycemia, development of mild preeclampsia at 32 weeks − **Case 3**: 3 consecutive pregnancies in one patient − Pregnancy no. 1 + 2: good metabolic control, pregnancy no. 3: frequent asymptomatic hypoglycemia − size of adenomas remained stable	− Case 1: cesarean section (no progress of labor, fetal tachycardia) − Case 2: cesarean section in 35th week of gestation (worsening of preeclampsia) − Case 3: − Pregnancy 2: cesarean section at 35 weeks for failed induction of labor − Pregnancy 3: planned cesarean at 35 weeks	− Case 1: uneventful − Case 2: FGR of one child (transfusor‐transfused‐syndrome) − Case 3: − Pregnancy 1: fetal death at 33 weeks (reason unclear) − Pregnancy 2 + 3: uneventful	Yes (1/7)
Ymamoto, Suzuki et al. 2010 [7]	**1 patient** − Severe preeclampsia after 20 weeks gestation (hypertension and proteinuria) − Hypoglycemia	− cesarean section at 26 weeks of gestation due to FGR and AEDF	− FGR − Death of newborn 2 days after birth (disseminated intravascular coagulation)	Yes
Lewis, Scrutton et al. 2005 [8]	**1 patient** − Pre‐existing hypertension and proteinuria, did not worsen − Stable lactate and glucose levels − No progression of existing adenomas	− Induction of labour at 38 weeks of gestation, vacuum delivery − (abnormal CTG) − blood transfusion due to hemorrhage	− Birth weight 10th percentile, uneventful	No
Lee, Muiesan et al. 2004 [9]	**1 patient** − Pre‐existing hypertension and proteinuria − Liver and kidney transplantation before pregnancy − Uneventful pregnancy	− n.a.	− Unexplained FGR	Yes
Jones, Tower et al. 2021 [11]	**1 patient** − No history of proteinuria or hypertension − Liver adenoma remained stable − Uneventful pregnancy	− Induction of labor at 37 weeks − Vacuum delivery (abnormal CTG)	− Transient hypoglycemia − Uneventful	No

Abbreviations: AEDF, absent end diastolic flow; CTG, cardiotocograph; FGR, fetal growth restriction.

^a^ No information about the subtype of GSD.

It is well known that the placenta plays a crucial role in the development of preeclampsia. In our case series, both patients with GSD Ia developed preeclampsia, and both had different but significant sonomorphological and histopathological anomalies of the placenta.

The changes in the placenta in the first case fit a mild preeclampsia, showing for example, acceleration of villi maturation and inter‐ and perivillous fibrin deposition [12]. There are many parallels to the case reported by Yamamoto et al. [7]: his patient with GSD Ia also developed preeclampsia and, as in our first case, the placenta was very small (155 g; 3rd percentile [19]). Histologically, in both cases there were subchorionic pseudoinfarctions with calcification and infarctions, typical for preeclampsia (Table 1).

Changes as inter‐ and perivillous fibrin deposition were also present in the placenta of our second patient. However, in this placenta further signs, for example, arrest of villous maturation, persisting embryonic stroma, and thickness of the placenta, predominated. These signs have been associated with a diabetic metabolic state [13, 20, 21] and may reflect the fluctuation of the patient's blood glucose levels with not only hypoglycemia but also often increased blood glucose concentrations. The intermittent polyhydramnion may reflect a hyperglycemic state.

It is known that preeclampsia is more frequent in conditions of microvascular disease like hypertension or diabetes [22].

The second patient was diagnosed to suffer from essential hypertension. Hypertension was well controlled during the whole pregnancy. There are no specific changes in the placenta for hypertensive diseases in pregnancy. There are combinations of findings (see table, section on maternal vascular malperfusion) that are mainly characteristic of forms of the disease with hypertension, but are not diagnostically conclusive.

According to the literature, these changes also occur in healthy pregnant women. In both placentas (case 1 and case 2), such stigmata were found that were focal and did not indicate clinically relevant damage to the placenta/infant due to maternal hypertension. There was no convincing evidence of maternal spiral artery damage caused by hypertension in the placental tissue.

Maternal hyperglycemia during pregnancy can cause structural changes in the placental vascularization [13], which can affect fetal development and growth [21]. However, previous studies found it difficult to establish a clear correlation between maternal metabolic disorders and various changes in the placenta, which may be attributable to differences in the type of metabolic disorder, glycemic control of the patients, and varying study protocols [13]. Furthermore, in addition to hypoglycemia and hyperglycemia, there are other metabolic factors like dyslipidemia, particularly hypertriglyceridemia, that were identified as a component of the preeclampsia disease process [23, 24]. Hypertriglyceridemia could have been one of the reasons for the development of preeclampsia in the second patient.

To our knowledge, no data are available on the effect of hypoglycemia on placentation or vascularization of the placenta. Recurrent hypoglycemia, including clinically unrecognized hypoglycemia common in GSD Ia, leads to increased endogenous glucose production by the fetus from the 2nd trimester onwards through increased gluconeogenesis, increased protein catabolism, and therefore decreased fetal growth [25]. The increase in carbohydrate requirements, especially early in pregnancy, additionally puts patients with GSD Ia at risk to develop severe hypoglycemia, possibly leading to impaired growth of the unborn child [4]. Therefore, self‐monitoring by CGM should be standard of therapy throughout the whole pregnancy in GSD Ia patients.

The first placenta showed relatively extensive histological changes as seen in placentas of FGR children (e.g., accelerated villous maturation, subchorionic pseudoinfarctions, and decreased size of basal plate). The cause of placenta derived growth restriction is thought to be decreased remodeling of the spiral arteries with decreased placental supply in early pregnancy. Decreased villi surface leads to decreased oxygenation, resulting in stress and increased production of interleukins, non‐infectious villitis, and altered end‐differentiation of vascular smooth muscle cells [26]. The first patient had a history of miscarriages, which is also indicative of (repeated) implantation failure.

In summary, histological examination of the placenta of the first patient reflected the predominant preeclampsia, and in the second patient, the impact of the poor metabolic state on the placenta was uncertain. The relationships between placentation, metabolic status, SGA/FGR, and the development of preeclampsia are very complex and far from being fully understood. In our case series, histological examination did not reveal any features of the placentas that were specifically attributable to GSD Ia. One reason for this could be that both patients were on dietary therapy and thus the possible effects of their disease could be mitigated or completely masked. Another important point is that the placenta of a metabolically healthy child was examined in affected mothers. Neither child had a glucose metabolic disorder itself. It would be interesting to examine the placenta of newborns affected by GSD Ia in further studies in order to investigate not only the effects of GSD Ia on the placenta from the mother's side but also from the newborn's.

It is likely that GSD Ia and associated metabolic changes have an impact on the placental structure. Sonomorphologic and macroscopic changes (Table 1) in the placenta existed in both cases and can be attributed to metabolic disorders, but as they were nonspecific and not very pronounced, further studies will be needed to work out the histological impact of GSD Ia on the placenta in more detail.

## 4 Limitations

The major limitation of our work is the small number of cases.

However, it should be noted that GSD Ia is a very rare condition. To our knowledge, we are the first to focus on placental changes in mothers with GSD Ia. Both patients developed pre‐eclampsia, and both placentas showed different but significant sonomorphological and histopathological changes. Although in both cases the fetuses showed FGR, it must be emphasized that the causes of FGR as well as the causes for preeclampsia are multifactorial.

## 5 Conclusion

In this case study, we report the cases of two primipara with GSD Ia. In patients with this inborn error of carbohydrate metabolism, preeclampsia and FGR may arise. The development of preeclampsia as well as the occurrence of FGR is multifactorial. Pregnancies of patients with GSD Ia can be complicated by blood glucose disorder. Especially, hypoglycemia and possibly pre‐existing vascular damage might pave the way for the development of preeclampsia and/or FGR. Further research is needed to better understand the underlying mechanism, especially in altered placental morphology, to tailor a specialized multidisciplinary supervision and care for pregnant women with rare metabolic disorders like GSD Ia.

## Author Contributions

All authors contributed to relevant aspects of the planning, implementation and reporting of the work described in the article. Eva Mildenberger and Meike A. Busch in particular contributed to the neonatal aspects of the work. Julia B. Hennermann and Frauke Lang are the experts who contributed to the metabolic aspects. Larissa Seidmann was responsible for the expertise from the pathological side. Doris Macchiella and Valeria Laufs examined the patients prepartum and were responsible for the obstetric part. Annette Hasenburg, Head of the Gynaecological Clinic, facilitated the preparation of the paper and contributed corrections.

## Consent

All procedures followed were in accordance with the ethical standards of the responsible committee on human experimentation (institutional and national) and with the Helsinki Declaration of 1975, as revised in 2000 (5). Informed consent was obtained from all patients for being included in the study.

## Conflicts of Interest

Frauke Lang has received payment of the fee by Vitaflo Deutschland GmbH to a private account for being Speaker at VIA Symposium Frankfurt 09‐20‐2024 (Topic GSD Type I). She has received payment of travel expenses, accommodation and participation fee at the SSIEM Meeting 2024 by Nutricia metabolics Deutschland GmbH. She received honorary as 1st Chairwoman of the Pediatric Dietetics Working Group and is a member of patient organization Glycogenosis Deutschland e.V. Julia B. Hennermann has received payment to the institution from Sanofi, Biomarin, Takeda, Amicus and Chiesi. For consulting fees, she has received payments to the institution and to herself from Chiesi, Takeda and Sanofi. Payment or honoraria for lectures, presentations, speakers bureaus, manuscript writing or educational events she has received to the institution from Amicus, to the institution and to herself from Sanofi, Chiesi and Takeda and to herself from Immedica. She received Support for attending meetings and/or travel from Amicus Therapeutics, Chiesi and Nutricia Metabolics. For Participation on a Data Safety Monitoring Board or Advisory Board, payments to the institution were made by Amicus, Sanofi, Chiesi and Takeda. Annette Hasenburg has received Consulting fees from AstraZeneca, GSK, LEO Pharma, Lilly, MSD PharmaMar, Roche, Pfizer, Streamedup!GmbH and Tesario. She has recived payments from AstraZeneca, Art Tempi, Celgen, Form GmbH, GSK, LEO Pharma, Lilly, MedConcept GmbH, Med update GmbH, Medical Event Solution, Pfizer, PharmaMar, Pierre Fabre Pharma, Roche Pharma, Streamedup!GmbH and Tesario for lectures, presentations, speakers bureaus, manuscript writing or educational events. AstraZeneca, Amgen, Daiiji, Eisai, GILEAD, Lilly, MSD, Pierre Fabre, Pfizer, Roche Pharma and Seagen have sponsored educational events to institution. Annette Hasenburg has unpaid roles in Leadership ESGO Task Force Psychoonkologie since 2011 and AGO (Arbeitsgemeinschaft Gynäkologische Onkologie) since 2022. She is a member in the following societies: Deutsche Gesellschaft für Gynäkologie und Geburtshilfe e.V. (DGGG), Deutsche Krebsgesellschaft (DKG), Deutscher Ärztinnenbund, Arbeitsgemeinschaft Gynäkologische Onkologie, Organkommission Ovar (AGO‐OVAR), Studienleitgruppe der Arbeitsgemeinschaft Gynäkologische Onkologie (AGO‐SLG), International Psycho‐Oncology Society (IPOS), Federation International of Gynecology and Obstetrics (FIGO), European Society of Gynecological Oncology (ESGO). AstraZeneca, Biontech, MSD, Novartis Pharma, Roche Pharma AG, AGO Research GmbH, Amgen, Daiiji, Eisai, GBG, GILEAD, IRCCS‐Instituto di Ricerche, Karyopharm Therapeutics Inc., Lilly, Novartis Pharma, Palleos healthcare GmbH, Roche Pharma Seagen, Tesaro, TRIO Canada, NOGGO Berlin funded research to institution. The remaining authors declare no conflicts of interest.

## Data Availability

The authors confirm that the data supporting the findings of this study are available within the article [and/or] its supplementary materials.
